# Antimicrobial Activity of Chitosan Derivatives Containing *N*-Quaternized Moieties in Its Backbone: A Review

**DOI:** 10.3390/ijms151120800

**Published:** 2014-11-13

**Authors:** Alessandro F. Martins, Suelen P. Facchi, Heveline D. M. Follmann, Antonio G. B. Pereira, Adley F. Rubira, Edvani C. Muniz

**Affiliations:** 1 Department of Chemistry, Universidade Estadual de Maringá (UEM), Av. Colombo, 5790, Maringá-PR 87020-900, Brazil; E-Mails: afmartins50@yahoo.com.br (A.F.M.); hevelinefollmann@hotmail.com (H.D.M.F.); guilebasso@hotmail.com (A.G.B.P.); afrubira@uem.br (A.F.R.); 2 Universidade Tecnológica Federal do Paraná (UTFPR)–Estrada para Boa Esperança, Dois Vizinhos, Paraná 85660-000, Brazil; E-Mail: ptbon_suh@hotmail.com

**Keywords:** chitosan, chitosan derivatives, quaternization, antimicrobial activity, antimicrobial mechanism

## Abstract

Chitosan, which is derived from a deacetylation reaction of chitin, has attractive antimicrobial activity. However, chitosan applications as a biocide are only effective in acidic medium due to its low solubility in neutral and basic conditions. Also, the positive charges carried by the protonated amine groups of chitosan (in acidic conditions) that are the driving force for its solubilization are also associated with its antimicrobial activity. Therefore, chemical modifications of chitosan are required to enhance its solubility and broaden the spectrum of its applications, including as biocide. Quaternization on the nitrogen atom of chitosan is the most used route to render water-soluble chitosan-derivatives, especially at physiological pH conditions. Recent reports in the literature demonstrate that such chitosan-derivatives present excellent antimicrobial activity due to permanent positive charge on nitrogen atoms side-bonded to the polymer backbone. This review presents some relevant work regarding the use of quaternized chitosan-derivatives obtained by different synthetic paths in applications as antimicrobial agents.

## 1. Introduction

### 1.1. Infections Caused by Microorganisms

Infections by microorganisms, such as gram-positive and gram-negative bacteria, virus, fungi, and protozoa, *etc.*, are major concerns in clinical and pharmaceutical areas (drugs, medical devices, odontology, hospital surfaces, *etc.*) as well as in the food industry (food packaging, storage, fresh products, *etc.*). The diseases caused by these microorganisms provoke serious health problems that in severe cases lead to death. Diseases related to the proliferation of microorganisms are particularly significant in hospitals where the risk of infection by microorganisms is a major concern, mainly when complicated surgical procedures are conducted. However, illnesses caused by poor personal hygiene and rotten or contaminated food should also be considered an important issue [1,2,3]. Therefore, the development of materials that exhibit antimicrobial activity appears to be highly relevant in health care. According to Musumeci *et* *al.* [4] an antimicrobial agent is a “substance that kills or inhibits the development and the multiplication of microorganisms, such as bacteria, fungi, protozoa or viruses”. Among numerous materials having this feature, chitosan and its derivatives can be highlighted. In what follows, some results related to the bacterial activity of chitosan and chitosan-derivatives are presented.

### 1.2. Chitosan and Chitosan Derivative-Based Materials and Their Bactericidal Activity

Over 1140 articles were found with “chitosan” and “antimicrobial activity” as keywords for bibliographic research using the SCOPUS^®^ database, with 740 of these published after 2010, demonstrating the high level of interest in the chitosan biopolymer as an antimicrobial agent. Apart from chitosan, chitosan-derivatives [5] have also attracted lots of interest, because they must have or even surpass some of the attractive properties observed in chitosan, especially regarding its bactericidal property against several types of bacteria [5,6]. Chitosan is a “partially deacetylated derivative of chitin, consisting of *β*-(1,4)-2-amino-2-deoxy-d-glucopyranose and small amounts of *N*-acetyl-d-glucosamine” [7]. Chitosan-derivatives are usually obtained by chemical modification of the amino or hydroxyl (especially at C6 position in the chitosan backbone) groups of chitosan for improving the physicochemical properties [7,8]. Chitosan and chitosan-derivatives have been extensively used to obtain polyelectrolyte complexes, due to their polycationic nature and their biological properties (biodegradability, biocompatibility, low toxicity, mucoadhesivity and antimicrobial) [9,10,11]. The literature mentions the bacterial activity of these materials on the basis of their physicochemical properties (molecular weight, hydrophilic/hydrophobic, water-solublility, positive charge density, degree of deacetylation, concentration, chelating capacity, pH, *etc.*) [2].

Some authors reported the bactericidal activity of chitosan-derivatives is stronger than that of unmodified chitosan. Jia *et* *al.* [12] showed the *N*-propyl-*N*,*N*-dimethyl chitosan presents bactericidal activity against *Escherichia* *coli* (*E*. *coli-*ATCC 25925) 20 times higher than that of chitosan with 96% deacetylation of *M*_v_ 2.14 × 10^5^, 1.9 × 10^4^ and 7.8 × 10^3^. Other authors reported that the antimicrobial activity of *N*,*N*,*N*-trimethyl chitosan (TMC) is ca. 500 times higher than that of unmodified chitosan. It has been shown that other chitosan-derivatives such as hydroxypropyl chitosan, *O*-hydroxyethylchitosan, and carboxymethyl chitosan, among others, also exhibit significant antimicrobial activity [7,12,13,14].

Several studies about the antimicrobial characteristics of films made of chitosan and its derivatives have been reported [15,16,17,18,19]. Such films exhibit strong antimicrobial activity against a variety of pathogenic and spoilage microorganisms, showing the efficiency of chitosan-based materials on bactericidal activity. Follmann *et* *al.* [19] developed TMC/heparin thin films using layer-by-layer (LbL) procedures on a chemically modified polystyrene surface (oxidized polystyrene surface) that presented antimicrobial and anti-adhesive properties against *E.* *coli* (ATCC 26922). The antibacterial property was dependent on the degree of quaternization and pH of the assays. Sun *et* *al.* [15] investigated the antimicrobial activity against *E.* *coli* (ATCC 43895), *Salmonella* *typhimurium* (ATCC 19585), *Listeria* *innocua* and *Bacillus* *subtilis* (ATCC 1254) on chitosan films with gallic acid at different concentrations. They found the addition of gallic acid increased the antimicrobial activities of the chitosan films. The results showed the strongest antimicrobial action on films with 1.5 g/100 g of gallic acid and the films may have the potential for applications in the health-care field.

Similarly, antibacterial polymers may also be incorporated into membranes, fibers, hydrogels, and beads, and used in several applications in the field of health, as for instance in wound dressing, tissue engineering, and drug delivery carriers, among others [2,20,21,22,23,24,25,26,27]. For example, chitosan acetate complexed with C12–C18 alkyl starch prophyl dimethylamine betaine (AAPDB) was evaluated against several microorganisms (*E.* *coli* (ATCC 25922), *Pseudomonas* *aeruginosa* (ATCC 27853), *Staphylococcus* *aureus* (ATCC 25923), *Staphylococcus* *epidermidis* and *Candida* *albicans*). It was observed that the chitosan/AAPDB complex showed strongest inhibitory effect for all the studied microorganisms when compared with unmodified chitosan and AAPDB [6]. Another work showed that the chemical modification of chitosan through heterocyclic substitution and further quaternization allows the product to present an important effect in the antimicrobial activity against microbes (gram-negative and gram-positive bacteria) and fungi. The derivatives prepared in that work showed significant inhibition against *Mycobacterium* *smegmatis* (MTCC 943) and *Pseudomonas* *aeroginosa* (MTCC 4676) at concentration ≈ 500 ppm, while the unmodified chitosan was not effective in the same concentration [28]. Fajardo *et* *al.* [29] studied the incorporation of silver sulphadiazine (AgSD) in chitosan/chondroitin sulfate (CS) matrices and performed antibacterial studies against *Pseudomonas* *aeruginosa* (ATCC 27853) and *Staphylococcus* *aureus* (*S.* *aureus* (ATCC 25923)) bacteria as well as cellular assays using VERO cells (healthy cells obtained from African green monkey kidney). The authors found that both matrices (chitosan/CS and chitosan/CS/AgSD) exhibit activity against *P.* *aeruginosa* and *S.* *aureus*, and had no toxic effects on VERO cells, which makes the use of chitosan/CS and chitosan/CS/AgSD even more attractive.

All these studies, based on chitosan and chitosan-derivative activities against microorganism, clearly indicate the diversity and relevance of the research and use of chitosan and its derivatives as antimicrobial agents.

## 2. Synthesis and Antimicrobial Property of Chitosan Derivatives without the Presence of *N*-Quaternized Nitrogen Atoms in Polysaccharide Structure

Chitosan antimicrobial activity depends on various factors, such as concentration, deacetylation degree, molecular weight and the solvent used [30,31,32,33,34]. The pH of chitosan solution is a factor that also influences the microbial activity of this polysaccharide [5]. The precise model for chitosan bactericidal action is still not fully elucidated, but some mechanisms have been proposed [19]. Chitosan presents positive charges density when the pH is lower than its p*K*_a_ (6.5). In this case, the protonated amino groups (NH_3_^+^) at the C2 position in the glucose monomer of chitosan chains allow the formation of a polycationic structure, which can interact with anionic compounds and macromolecular structures of bacteria [1,35]. This interaction can alter bacterial surface morphology, increasing membrane permeability and promoting leakage of intracellular substances (e.g., proteins including lactate dehydrogenase, nucleic acids and glucose), or even decrease membrane permeability and, consequently, repress nutrient transport [1,36]. Some studies have confirmed the occurrence of the increased permeability and disruption of cell membranes. It was postulated that positively charged chitosan containing protonated NH_3_^+^ sites interacts with cellular DNA, allowing chitosan transport into the cells, thereby inhibiting transcription [36]. On the other hand, the use of chitosan in biological applications is restricted, due to its low solubility at neutral pH. Therefore, much effort has been made to prepare chitosan-derivatives that are soluble in water, especially at physiological pH [37,38].

The chitosan-derivatives free of quaternization have good solubility in aqueous solution at neutral pH and present excellent antimicrobial activity at this condition. This review initially discusses the synthesis of chitosan-derivatives free of *N*-quaternized groups, from different synthetic methodologies. However, the main focus was to describe some recent synthetic methodologies to obtain chitosan-derivatives containing quaternized moieties in their backbone. These derivatives of chitosan present excellent antimicrobial activity at neutral condition (pH ≈ 7) and good potential for applications in the medical and pharmaceutical field. Finally, this review discusses the construction of chitosan-based materials containing quaternized moieties in their structures. The antimicrobial mechanism of such materials will be addressed throughout each section.

### 2.1. Modification of Chitosan Mediated by Carbodiimide as Reactant

**Scheme 1:**
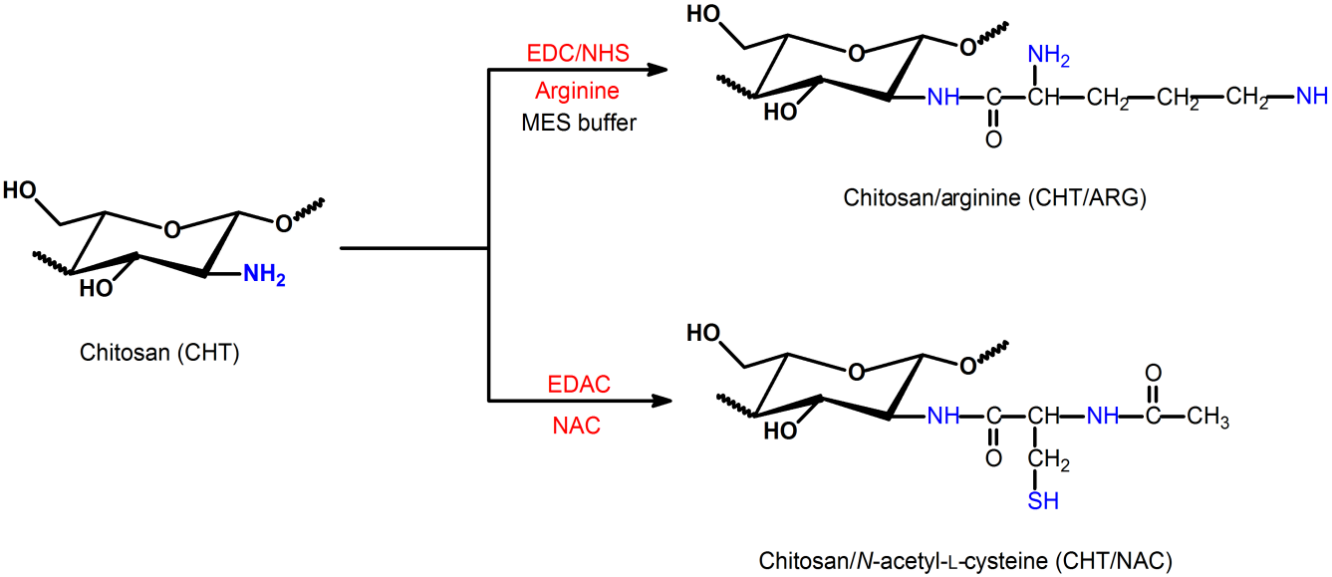
Route for chitosan/arginine (CHT/ARG) and chitosan/*N*-acetyl-l-cysteine (CHT/NAC) preparation using 1-ethyl-3-(3-dimethylaminopropyl) carbodiimide hydrochloride (EDC)/*N*-hydroxysulfosuccinimide sodium salt (NHS) in 2-(*N*-morpholino) ethanesulfonic acid sodium salt buffer solution (MES) and *N*-(3-dimethylaminopropyl)-*N*'-ethylcarbodiimide hydrochloride (EDAC), respectively [43,44].

Nowadays, there are several reports in the literature about modification of chitosan using carbodiimide as reagent [39,40,41,42]. For example, arginine (ARG) functionalized chitosan-derivatives were obtained through reaction with 1-ethyl-3-(3-dimethylaminopropyl) carbodiimide hydrochloride (EDC), using *N*-hydroxysulfosuccinimide sodium salt (NHS) as a catalyst agent in 2-(*N*-morpholino) ethanesulfonic acid sodium salt buffer solution (MES) (Scheme 1) [43]. Other chitosan-derivatives were obtained from *N*-(3-dimethylaminopropyl)-*N*'-ethylcarbodiimide hydrochloride (EDAC) [44]. In this case, *N*-acetyl-l-cysteine (NAC) functionalized chitosan was obtained (Scheme 1). Li *et* *al.* [39,40] developed biodegradable and biocompatible chitosan derivatives grafted with poly (lactic acid) using EDC and NHS to activate carboxyl groups of lactic acid.

Chitosan/ARG with various substitution degrees (DS) from 6.0% to 30% were prepared by reacting amino groups of chitosan with arginine [44]. These chitosan-derivatives are highly soluble in water, since the p*K*_a_ of the guanidinium side chain of arginine is around 12.5. Thus, chitosan/ARG derivatives present positive charge density at neutral pH environments [43].

Tang *et* *al.* [44] reported the antibacterial activity of chitosan/arginine derivative against gram-negative bacteria *Pseudomonas* *fluorescens* (*P.* *fluorescens* (ATCC 700830)) and *E.* *coli* (ATCC 25922) and the microbial action mode. They found chitosan had antibacterial activities only at acidic medium, due to its low solubility at pH > 6.5. So, chitosan/arginine, soluble at pH ≈ 7.0, indicated that both substituted derivatives with DS = 6% and 30% inhibited significantly *P.* *fluorescens* and *E.* *coli* growth up to 24 h at concentrations ≥ 128 mg L^−1^ for *P.* *fluorescens* and ≥ 32 mg L^−1^ for *E.* *coli*. Studies using fluorescent probes and field emission scanning electron microscopy (FESEM) showed chitosan/arginine antibacterial activity is, mainly, due to the increase of membrane permeability, a fact attributed to interaction between chitosan/ARG derivative and the bacteria [44]. Chitosan/arginine promotes 1-*N*-phenylnaphthylamine (NPN) uptake at pH ≈ 7 and it is likely that NPN uptake occurs through a similar mechanism upon exposure to either modified or unmodified chitosan polymers. The main advantage of a chitosan/arginine derivative is its polycationic feature at physiological pH. NPN is a hydrophobic fluorescence probe widely used to assess cell membrane permeability, since its quantum yield increases greatly in hydrophobic environments compared to aqueous environments [44].

**Figure 1:**
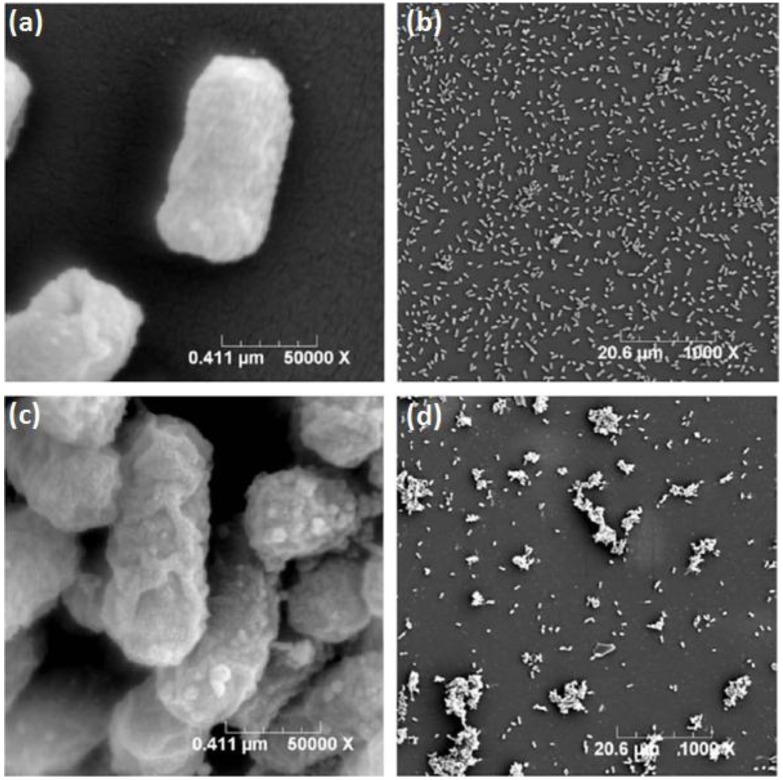
SEM images of *E.* *coli* after incubation with 100 mg L^−1^ chitosan/arginine (CHT/ARG) for 3 h. Controls (**a**–**d**); cells treated with 6%-substituted CHT/ARG (**b**) and cells treated with 30%-substituted CHT/ARG (**c**). Reprinted with permission from reference [44]. Copyright 2010 Elsevier.

Under normal conditions, NPN is excluded by the outer membrane (OM) barrier of gram-negative bacteria. According to Tang *et* *al.* [44] when the OM structure is damaged, NPN can partition into the hydrophobic interior of the OM, or plasma membrane, leading to a dramatic increase of its fluorescence. Therefore, the increase of NPN fluorescence intensity promoted an increase of cell membrane permeability. The OM contains polyanionic lipopolysaccharides (LPS) stabilized by divalent cations, such as Mg^2+^ and Ca^2+^. Thus, due to the chelating ability of chitosan and some chitosan-derivatives, the divalent metal ions bound to LPS and proteins form chelates with chitosan-based materials. Based on this kind of interaction, the cell walls of bacteria will become more volatile, leading to the leakage of cytoplasm constituents and resulting in the death of bacteria [1,45]. The OM acts as a permeability barrier and inhibits the transport of macromolecules and hydrophobic compounds entering or leaving bacteria cell membranes [45]. The cation-binding sites maintain the LPS stability and are essential to OM integrity. However, cationic molecules such as chitosan and some chitosan-derivatives could interact with divalent cations bound to LPS that maintain the integrity of the bacterial membrane, while promoting disorganization of OM structure. From FESEM analysis cell aggregation was observed for both *E.* *coli* (ATCC 25922) and *P.* *fluorescens* (ATCC 700830), immediately after the addition of the chitosan/arginine derivative [43,44], and *E.* *coli* cells remained unlysed after the chitosan/arginine treatment (Figure 1). So, the chitosan/arginine derivative increased cell membrane permeability, due to interaction of the polycationic derivative with the *E.* *coli* cell membrane.

Xiao *et* *al.* [43] studied the bactericidal action of chitosan/arginine on *S.* *aureus* (CCTCC AB910393) a gram-positive bacterium. In this case, the antibacterial effect is different from that on *E.* *coli* (CCTCC AB91112), a gram-negative bacterium, which may be ascribed to its different cell wall structure. In gram-positive bacteria, the cell wall is composed of a broad dense wall that consists of 15–40 interconnecting layers of peptidoglycans. Positively charged free –NH_3_^+^ or/and guanidine groups of chitosan or chitosan/arginine can bind tightly to the cell wall components, resulting in pore formation in the cell walls, causing severe leakage of cell constituents and eventually the death of the cell. When concentrations of chitosan and chitosan/arginine decrease, they are not able to destroy the cell walls by distortion-disruption and, instead, chitosan or chitosan/arginine are digested and adsorbed by bacteria as nutrition to accelerate the growth of the microbes. In addition, it was found that chitosan-derivatives with more positive charges possess decreasing antibacterial activity against *S.* *aureus*. These results may imply that the higher cationic charge is not, by itself, responsible for better antimicrobial activity than that of unmodified chitosan [43].

Fernandes *et* *al.* [45] evaluated the effect of antimicrobial activity of the chitosan/*N*-acetyl-l-cysteine complex (prepared from carbodiimide-mediated reaction) on *E.* *coli* (CECT 101) and *S.* *aureus* (CECT 86). The Langmuir monolayer technique was applied to elucidate the interactions of the chitosan and chitosan/*N*-acetyl-l-cysteine with the bacteria membrane using a cell membrane model. The anionic phospholipid dipalmitoylphosphatidylglycerol (DPPG) is a major component of gram-negative and gram-positive bacteria [45]. This negatively charged phospholipid interacts with the primary amines and sulfhydryl groups, which are believed to strongly account for its antibacterial activity. In this case, the microbial activity of thiolated-chitosan was demonstrated to be primarily due to electrostatic interactions with DPPG, but also due to the uncharged amino and sulfhydryl groups of the biopolymer and/or the specific conformation of its macromolecules in solution [1,45].

### 2.2. Methylation Process of Schiff Bases

**Scheme 2:**
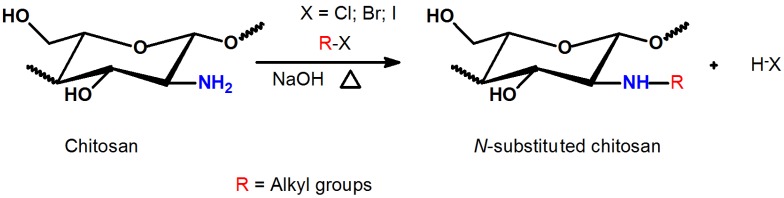
Reaction used for synthesis of *N*-substituted chitosan derivatives by halogen displacement reaction [46].

**Scheme 3:**
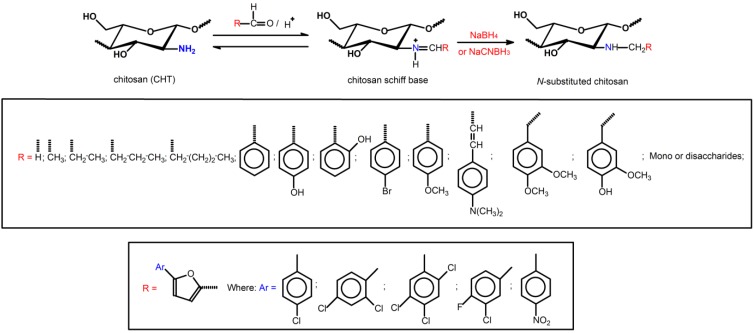
Route for synthesis of *N*-substituted derivatives from chitosan Schiff base followed by reductive amination reaction [46,48,49].

The direct *N*-substitution of chitosan can be processed through reaction among the primary amino groups and alkyl halides, in the presence of a strong base under heterogeneous conditions (Scheme 2) [46]. This reaction is performed in vigorous conditions, such as high sodium hydroxide concentration and elevated temperature, resulting in molecular depolymerization and chitosan-derivatives with low substitution degree (DS = 24.5%) [46,47]. On the other hand, selective *N*-alkylation and *N*-arylation were performed via Schiff base intermediates [46,48,49]. The reaction was achieved, reacting the primary amino groups on chitosan backbone with some aldehydes, under homogeneous acidic conditions, followed by Schiff base reduction with sodium borohydride or sodium cyanoborohydride [46] (Scheme 3).

Scheme 3 lists a series of *N*-substituted chitosan-derivatives obtained from Schiff base reduction. Increased antimicrobial activity of the *N*-alkylated chitosan-derivatives, containing disaccharides (maltose, lactose and cellobiose) as *R* groups, against *E.* *coli* (CCRC 10675) as the pH changed from 5.0 was observed and reached maximum at pH ranged from 7.0 to 7.5 [49]. The possible explanation for this is that the chitosan *N*-alkylation with disaccharide changed the p*K*_a_ of the chitosan-derivative compared to chitosan, which made the protonation of chitosan-derivative molecules different from their precursor (chitosan) under similar pH conditions. Guo *et* *al.* [50] studied the antifungal activity of chitosan, Schiff bases of chitosan and *N*-substituted chitosan-derivatives against *Botrytis* *cinerea* (*B.* *cinerea*) and *Colletotrichum* *lagenarium* (*C.* *lagenarium*). In these cases, phenyl and 2-hydroxyphenyl were the aryl groups present in the Schiff bases and chitosan-derivatives (Scheme 3). The chitosan-derivatives presented good antifungal activity, however this property depends on pH. The insertion of alkyl and/or aryl groups increased the hydrophobic property of chitosan-derivatives. These characteristics enhanced the interaction with the cell membrane of microorganisms and improved the antimicrobial activity of the chitosan compounds [50,51]. This was observed due to the hydrophobicity of microorganisms cell membranes. However, it is also proposed that the antifungal activities of chitosan and chitosan-derivatives occur due to its polycationic property. Fungi microbial cells are negatively charged and, thus, the action mechanisms of chitosan and chitosan-derivatives on fungi microbial cells are similar to those previously reported by Guo *et* *al.* [51]. The polycationic property of these compounds increased the microbial action, however the chitosan and *N*-alkyl, *N*-aryl, *N*,*N*-alkyl and *N*,*N*-aryl substituted chitosan-derivatives presented poor solubility and low microbial action at physiological pH. Thus, it is evident that the positive charge density increases the bactericidal activity [7,8]. Therefore, to overcome the solubility limitation, a series of chitosan-derivatives containing *N*-quaternized groups were obtained from *N*-substituted compounds, as shown in Scheme 3. The improved solubility augments the application spectrum of these compounds and the existence of *N*-quaternized groups in the structure of chitosan-derivatives enlarges the antimicrobial activity at physiological pH [52,53]. In addition to being more soluble, these derivatives have hydrophobic groups (alkyl or aryl groups) in their structures that can enable better interaction with the microbial cells [19]. Therefore, the *N*-quaternized chitosan-derivatives present good hydrophilic-hydrophobic properties (which enables interaction with microbial cells that are hydrophobic and have surface negative charge density in their membranes) and, beyond this, are still soluble in neutral conditions, due to positive charge density [53]. The synthesis and antimicrobial activity of *N*-quaternized chitosan-derivatives will be addressed in section 4 of this review.

### 2.3. Other Methods

**Scheme 4:**
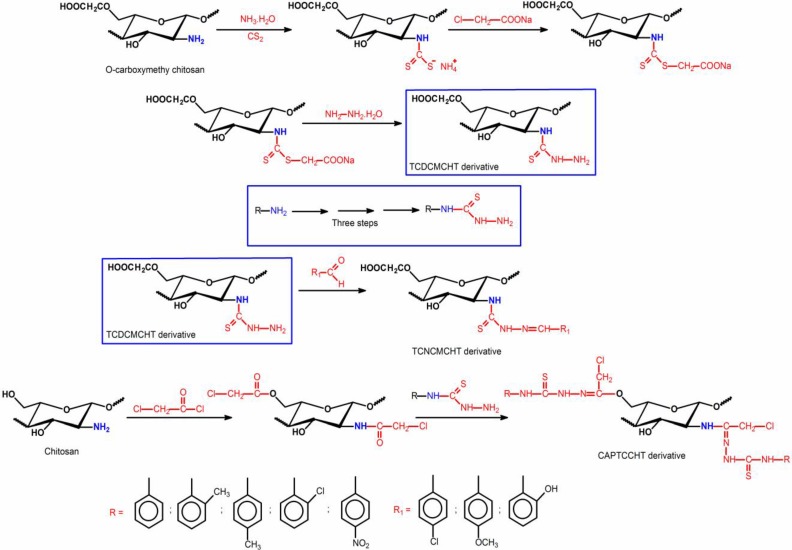
Routes for synthesis of thiosemicarbazide carboxymethyl chitosan (TCDCMCHT) and thiosemicarbazone carboxymethyl chitosan (TCNCMCHT) derivatives containing different *R*_1_ groups and synthesis of chloracetyl phenyl-thiosemicarbazone chitosan (CAPTCCHT) derivatives containing different *R* groups from chloracetyl chitosan (CACHT) derivative [56,57,58].

Thiosemicarbazones belong to an important class of compounds that contains nitrogen and sulfur atoms in their structures [54,55]. So, such compounds have donor bindings that are of considerable interest [56]. In order to investigate the antimicrobial activity of thiosemicarbazone chitosan-derivatives, Mohamed *et* *al.* [56] obtained three novel thiosemicarbazone *O*-carboxymethyl chitosan derivatives (TCNCMCHT) through condensation reaction of thiosemicarbazide *O*-carboxymethyl chitosan (TCDCMCHT) with *o*-hydroxybenzaldehyde, *p*-methoxybenzaldehyde and *p*-chlorobenzaldehyde (Scheme 4). In this case, *R*_1_ groups indicated the grafted aryl radicals in the TCNCMCHT derivatives.

Antimicrobial activity of such *O*-carboxymethyl chitosan-derivatives was evaluated against three bacteria lineage (*S.* *aureus-*RCMBA2004, *Bacillus* *subtilis* (RCMBA 6005) and *E.* *coli* (RCMBA 5003) and against three fungi lineage (*Aspergillus* *fumigates-*RCMBA 06002, *Geotrichum* *candidum* (RCMB 05098) and *Candida* *albicans* (RCMB 05035) [56]. Such compounds were more bactericidal for gram-positive bacteria than gram-negative bacteria. The inhibition indices of *o*-hydroxybenzaldehyde, *p*-methoxybenzaldehyde and *p*-chlorobenzaldehyde against *S.* *aureus* were 17.9 ± 0.36, 20.6 ± 0.27 and 24.3 ± 0.25, respectively. Compared to the other chitosan-derivatives, the *p*-chlorobenzaldehyde compound presented higher inhibition indices on *Bacillus* *subtilis* and on *E.* *coli* [56].

The results showed the TCDCMCHT and TCNCMCHT have higher antibacterial activity when compared to *O*-carboxymethyl chitosan and these chitosan derivatives presented good solubility compared to unmodified chitosan [56]. The introduction of thiosemicarbazide and thiosemicarbazone moieties onto *O*-carboxymethyl chitosan chains increases their cationic property; thus, their NH and C=S groups can be protonated and, consequently the net positive charge is strengthened, leading to a better antibacterial activity. Another proposed mechanism is the binding of TCDCMCHT and TCNCMCHT with microbial DNA, which promotes the inhibition of protein synthesis by the penetration of *O*-carboxymethyl chitosan derivatives into the nuclei of the microorganisms [56]. Thiosemicarbazide and thiosemicarbazone moieties grafted onto the hydrophilic *O*-carboxymethyl chitosan, decreased the intensity of H-bond interactions that prevailed in the unmodified chitosan. This explains why such derivatives penetrate easily into microorganism cell membranes, inhibiting the growth of the cell, since it inhibits the transformation of DNA to RNA [56].

The third mechanism is the chelation of metals, whereas the suppression of such species is essential for repressing the microbial growth [56]. It has been established that the –COOH, thiosemicarbazide and thiosemicarbazone groups have an excellent metal-binding capacity. This explains the higher antibacterial activity of TCDCMCHT and TCNCMCHT derivatives related to carboxymethyl chitosan, as previously described. Carboxymethyl chitosan derivatives showed higher activity against gram-positive bacteria than against gram-negative bacteria. The results also reveal that the antibacterial activity is affected by the nature of the substituent group (*R*_1_) found in the aryl ring of TCNCMCHT (Scheme 4). The chloride derivative is characterized by greater antibacterial activity than that of the hydroxyl and methoxy derivatives. According to Mohamed *et* *al.* [56], this may be attributed to the electron-withdrawing character of the chlorine group that decreases the electron density in the thiosemicarbazone group, increasing its cationic character.

A previous work reported by Zhong *et* *al.* [57] showed the preparation of acetyl and phenyl-thiosemicarbazone chitosan-derivatives and further evaluation of antimicrobial activities. The results also indicated that the antimicrobial action of the derivatives has a relationship with the grafted groups with different inductivity. In another study, Zhong *et* *al.* [58] obtained chloracetyl phenyl-thiosemicarbazone chitosan (CAPTCCHT) derivatives containing different *R*-substituent groups (Scheme 4). The correlation between the grafted group structure and antimicrobial activities was further evaluated. The results showed the antimicrobial activities of some derivatives were higher than unmodified chitosan. The antifungal and bactericidal actions of the synthesized compounds were related to the positive polarity of the N_4_ atoms and to the distribution of the electron atmosphere in the C=S groups. Antimicrobial activity was enhanced when the strong electron-donating group (–CH_3_) was present at the *p*-position of the phenyl in N_4_ and that activity decreased if a strong electron-withdrawing group (–NO_2_) was present at the same N_4_ position. These results demonstrate that the bioactivity of CAPTCCHT derivatives is affected by the positive polarity of the N_4_ atom and the distribution of the electron atmosphere in the C=S group [57,58].

Zhong *et* *al.* [58,59] and Mohamed *et* *al.* [56] observed different effects of microbial action for thiosemicarbazone derivatives (TCNCMCHT and CAPTCCHT). Besides the position and nature of the substituent on thiosemicarbazone chains, the structure of these derivatives should also affect the microbial action of the compounds (Scheme 4).

## 3. Synthesis of Chitosan Derivatives Containing *N*-Quaternized Nitrogen Atoms in Polysaccharide Structure and the Effect on Their Antimicrobial Action

Several studies have reported the antimicrobial activity of chitosan, *N*-monosubstituted and *N*,*N*-disubstituted chitosan-derivatives [43,50,51,60] at acidic media. However, due to the limited solubility of such materials and low microbial activity at physiological pH conditions, several research groups have transposed this barrier, obtaining chitosan-derivatives with *N*-quaternized groups [53,61,62,63] that are soluble in a wide pH range. As previously reported, the antimicrobial action depends mainly on polycationic portions of material structures and so there are efforts to obtain compounds with *N*-quaternized groups [63]. Such groups remain positively charged at any pH and may strongly interact with microorganism cell membranes; an effective antimicrobial inhibition then occurs at physiological pH [19,53]. *N*,*N*,*N*-trimethyl chitosan (TMC) was the first chitosan-derivative synthesized possessing *N*-quaternized sites [–^+^N(CH_3_)_3_] in its structure [59]. TMC has bactericidal activity, which is dependent on the degree of quaternization (DQ) [53,64]. Research groups are interested in obtaining water-soluble *N*-quaternized chitosan-derivatives with high contents of *N*-quaternized sites. These sites possess hydrophobic methyl groups that could increase the interaction with the lipid cell membrane, promoting improved antimicrobial activity [61,63,65]. The synthesis and the excellent antimicrobial activity of chitosan derivatives containing *N*-quaternized moiety are presented as follows.

### 3.1. Synthesis of N,N,N-trimethyl Chitosan (TMC) and Its Antimicrobial Activity

**Scheme 5:**
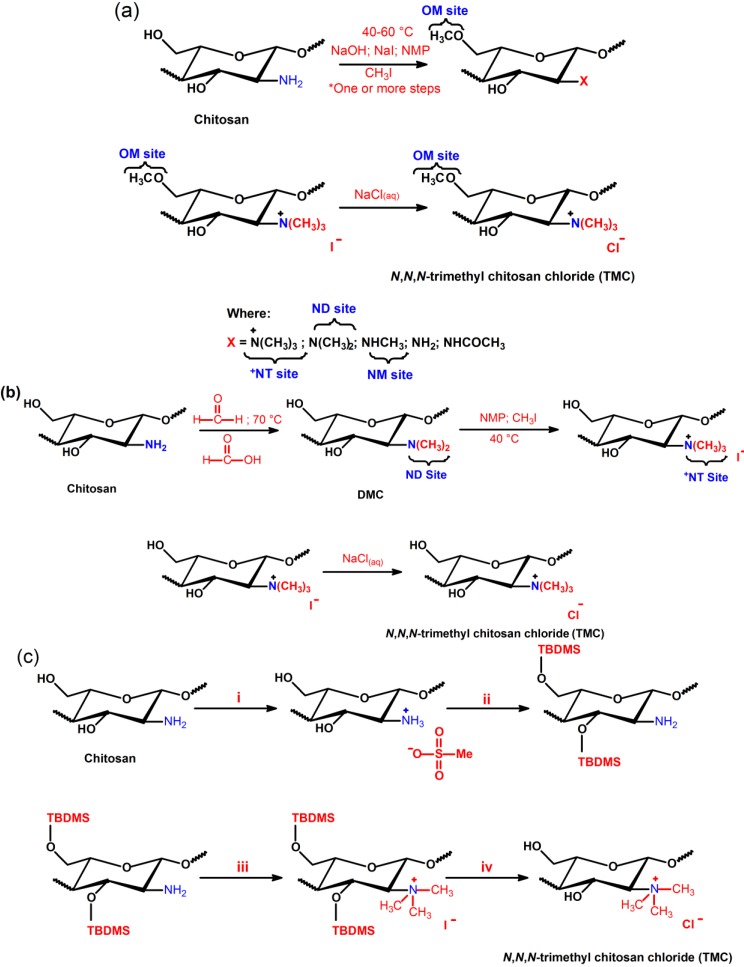
(**a**) Synthetic pathway for the preparation of *O*-methylated TMC according to the method of Sieval *et* *al.* [66]; (**b**) Two-step synthetic pathway for the preparation of TMC avoiding *O*-methylation according to the method of Verheul *et* *al.* [75]; and (**c**) Preparation of TMC fully *N*,*N*,*N*-trimethylated free of *O*-methylation. Reagents and conditions: (**i**) methanesulfonic acid, water, 10 °C; (**ii**) TBDMS chloride (5 equivalent), imidazole (10 equivalent), DMSO, N_2_, room temperature; (**iii**) CH_3_I (15 equivalent), Cs_2_CO_3_ (4 equivalent), NMP, 60 °C; and (**iv**) TBAF (1 mol L^−1^), NMP, 50 °C [76].

One method for preparing the *N*,*N*,*N*-trimethyl chitosan (TMC) is the use of iodomethane in alkaline solution of *N*-methyl-2-pyrrolidinone (NMP). The quaternization is performed from nucleophilic substitution of the primary amino group at the C2 position of chitosan with iodomethane and sodium iodide [66,67,68]. Muzzarelli *et* *al.* [59] obtained, for the first time, TMC salt, as shown in Scheme 5a. Consequently, many research groups synthesized TMC using the synthetic route described in such a scheme. The *N*-quaternization process depends primarily on the sodium hydroxide concentration, of the reaction time and of reaction steps [66]. The higher the reaction time and number of steps, the greater the degree of quaternization (DQ) of TMC will be [66,69,70,71]. The strongly alkaline medium promotes the methylation of the hydroxyl groups present in the C3 and C6 positions and also allows cleavage of glycosidic linkages [72]. Cleavage dramatically decreases the molar mass of TMC related to unmodified chitosan. TMC synthesis can be carried out in the temperature range of 40–60 °C. The initially synthesized *N*,*N*,*N*-trimethyl chitosan iodide salt is dissolved in aqueous NaCl solution (10% *w*_t_/*v*) and the exchange of iodide ion by chloride proceeds further. So, *O*-methylated *N*,*N*,*N*-trimethyl chitosan chloride salt with good solubility at physiological pH is obtained. However, the solubility depends on DQ and also on the degree of *O*-methylation, whereas the excessive *O*-methylation substantially reduces the respective chitosan-derivative solubility in water [66,67]. TMC with DQ = 52.5% and also containing *O-*methyl groups in the C3 and C6 position on chitosan chains has been synthesized using dimethylsulfate (another methylating agent) [73,74]. Dimethylsulfate is considerably less expensive and toxic when compared to the iodomethane [67]. In addition, it also has a high boiling point and no solvent is required for the reaction [73]. In the previously reported procedures, Bendiktsdottir *et* *al.* [74] concluded that the *O*-methylation process cannot be fully controlled and the resulting chitosan derivatives will always be a mixed heteromeric product which is a major drawback for structure-activity investigations [74].

Recently, Verheul *et* *al.* [75] synthesized TMC without *O*-methylation using only two steps, as shown in Scheme 5b. In the first step, the *N*-dimethylation of amino sites on the chitosan backbone, performed from formic acid-formaldehyde mixture (Eschweiler-Clarke) at 70 °C, was used to synthesize *N*,*N*-dimethyl chitosan (DMC) [75]. The quaternization of the DMC was achieved by using iodomethane in NMP at 40 °C without the presence of sodium iodide and sodium hydroxide. In this case, the *O*-methylation was not observed, and the molecular weight of *O*-methyl free *N*,*N*,*N*-trimethyl chitosan chloride salt slightly increased, compared to unmodified chitosan, implying that no chain scission occurred during synthesis, resulting in an increased DQ [75]. TMC free of *O*-methyl sites (OM groups) with DQ > 25% presented good solubility, while TMC *O*-methylated with DQ > 15% still showed solubility at physiological pH. *O*-methyl TMC exhibits high structural heterogeneity compared to TMC free of OM groups, since that presents in its structure *N*-methylated (NM sites), *N*,*N*-dimethylated (ND sites), *N*,*N*,*N*-trimethylated (^+^NT sites), *O*-methylated (OM sites) groups and at rest acetylated moiety, shown in Scheme 5a. On the other hand, TMC free of *O*-methylation does not possess OM and NM sites in its structure, because the methylation synthesis of TMC without OM sites was controlled, as shown in Scheme 5b. This structural difference alters significantly the physicochemical and biological properties of these derivatives [75].

More homogenously *N*-quaternized chitosan derivatives, in this case TMC free of *O*-methylation, could be synthesized by protecting the hydroxyl groups present at the C3 and C6 positions on the chitosan backbone (Scheme 5c) [74]. Group protection strategies have been used for the synthesis of chitosan-derivatives and, as consequence, these compounds present good solubility in organic solvents, because the H-bond intensities among chitosan chains decreased substantially [76]. Hydroxyl groups on chitosan backbone can be protected by di-*tert*-butyl-dimethylsilyl (di-TBDMS) groups, which present good stability under basic conditions and moderately acidic conditions, and can still be removed under strongly acidic conditions without affecting other functional groups [74,76]. TBDMS-protected chitosan was used as a precursor in the synthesis of fully trimethylated TMC and consequently TMC free of *O*-methylation was obtained (Scheme 5c) [76].

Furthermore, it has been shown that TMC derivatives can act as antibacterial agents on gram-positive and gram-negative bacteria [19,53]. The antimicrobial activities of TMC free of OM groups and *N*,*N*,*N*-trimethyl-*O*-carboxymethyl chitosan-derivatives against *S.* *aureus* (ATCC 6538) and *E.* *coli* (ATCCDH5α) were evaluated recently [53]. In this case, *N*,*N*,*N*-trimethyl-*O*-carboxymethyl chitosan acted more weakly than TMC, and its activity decreased as the degree of carboxymethylation increased, *i.e.*, the carboxymethylation did not directly enhance the antibacterial activity [53]. The results showed that the activity of ^+^NT groups was weaker than that of other non-quaternized amine groups (NM and ND sites) at pH ≤ 5.5. On the other hand, the antibacterial activity of TMC free OM sites increased with the DQ at pH ≥ 5.5. Xu *et* *al.* [53] and Follmann *et* *al.* [19] pointed out that the exchange of the chloride ions by hydroxyl anions in the *N*-trimethylated sites (^+^NT), from aqueous TMC solution, is favoured at pH ≥ 5.5 (Scheme 5), according to the equations

–NT^+^Cl^−^ ↔ ^+^NT + Cl^−^ (1)

–^+^NT + H_2_O ↔ NT^+^OH^−^ + H^+^ (2)

Those authors stated that the –NT^+^Cl^−^ groups could not interact with the negative charged sites on the cell envelope of *E.* *coli* and TMC95 (TMC with DQ = 95%) presented strong bactericidal activity only at pH 7.2, proving that the ^+^NT groups contributed to the antibacterial activity. At pH ≥ 5.5 the TMC dissociates to form *N*,*N*,*N*-trimethyl chitosan hydroxide. So, the dissociation and subsequent deshielding of the ^+^NT groups increased the bacteriostatic action [7,53]. Runarsson *et* *al.* [64] believed that the protonated or modified amine groups (^+^NH_3_, ^+^NM and ^+^ND sites) rather than the *N*-trimethylated ones contributed to the antibacterial activity, and the NM and ND groups functioned the same as the free –NH_2_ groups. The lower pH benefits the protonation of the NM and ND groups, but represses the ionization of NT^+^Cl^−^ sites. Meanwhile, because of the repulsive forces among ^+^NT groups and H^+^, the TMC chains curled more heavily than chitosan and its interaction with the cell envelope was reduced [19,53]. Therefore, compared with chitosan, the chains of TMC derivatives were more flexible and interacted more easily with the bacteria cell envelope at pH ≥ 5.5. So, TMC derivatives were more efficient than chitosan at physiological pH and the mechanism of microbial inhibition of TMC derivatives was similar to that proposed for chitosan [52,63]. According to reports, *N*-quaternary materials may be more promising than chitosan in areas where the use of neutral medium is necessary. Besides the factors already mentioned, others may also have influenced the obtained results, such as the methodology used for TMC synthesis (proportion of NM, ND and ^+^NT sites and ion exchange of TMC), and the assay conditions such as culture temperature and ion strength [53,64].

**Figure 2:**
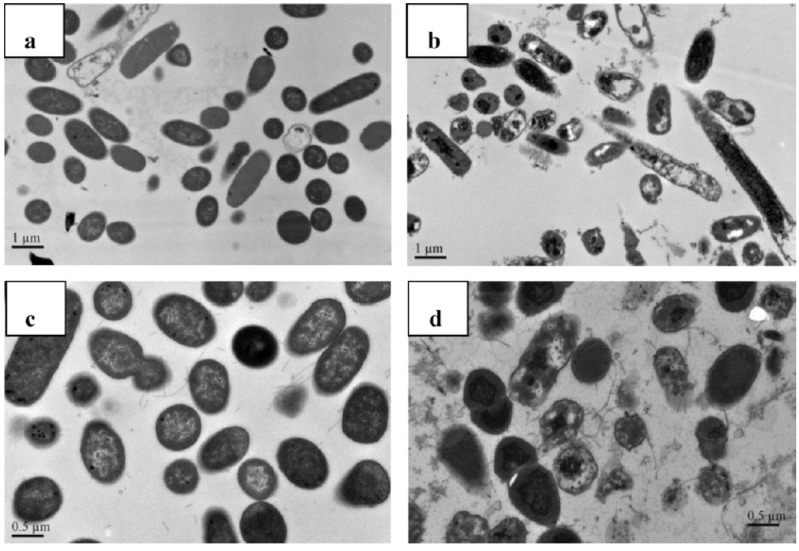
Effect on the cell morphology of *E.* *coli*: control (**a**); after interaction with TMC (**b**) and *S.* *aureus*: control (**c**); after interaction with TMC (**d**). Reprinted with permission from reference [77]. Copyright 2013 Elsevier.

Water-soluble quaternary chitin/chitosan chloride salt derivatives, *N*-[(2-hydroxy-3-trimethylammonium) propyl chitosan (NHT-chitosan), *O*-[(2-hydroxy-3-trimethylammonium)propyl chitin (OHT-chitin) and TMC, having an identical molecular weight, were prepared and their antibacterial activities against *E.* *coli* (CICC 21524) and *S.* *aureus* (CICC 10384) were evaluated at pH = 7.1 [77]. The results showed that TMC without OM sites exhibited better antimicrobial activity when compared to OHT-chitin and NHT-chitosan derivatives. It is necessary to highlight that the OHT-chitin and NHT-chitosan possess quaternary moieties, which are attached via four methylene long spacers to the polymeric backbone [77]. In terms of antimicrobial action, it works better when the positive charge (H^+^) is situated in the amine groups sided to the chitosan backbone. According to Huang *et* *al.* [77] the good antimicrobial activity is a synergistic effect of quaternization and chitin/chitosan backbone themselves. The bacterial morphology after contact with TMC was studied by TEM (Figure 2). As the *E.* *coli* and *S.* *aureus* were not treated with TMC, they remained intact and the usual surface cell morphology, without the release of intracellular components and the integrity of cell surface was maintained (Figure 2a,c). On the other hand, as treated with TMC free of OM groups cellular some changes on *E.* *coli* occurred, such as the regulation of the cytosolic components, even a few irregularly condensed masses were observed [77]. Above all, TMC-treated *E.* *coli* showed badly disrupted and altered cell membranes after 2 h. The results indicated that positively charged ^+^NT sites bound to the membrane leading to leakage of intracellular contents, such as glucose and lactate dehydrogenase, and ultimately causing the death of the *E.* *coli* cells (Figure 2b) [77]. For *S.* *aureus*, as the Figure 2d shows, some cells seemed to have condensed and other cells, which lost cytoplasmic materials, looked empty, although the overall cell shape was still recognizable and dark floccules surrounding the cells were observed, which contributed to the debris of lysed bacteria [77]. According to Huang *et* *al.* [77], soluble TMC could enter the cell without difficulty and intracellular materials appear to be more tightly packed despite lacking any organization, ultimately leading to the death of *S.* *aureus* cells (Figure 2d).

Since discoveries about the bactericidal activity of TMC derivatives, several research groups around the world have struggled to synthesize new derivatives containing different *N*-quaternized groups and larger amounts of their sites in the chitosan-derivative structures [28,78,79]. The goal was to find chitosan-derivatives with stronger bactericidal activity than TMC.

### 3.2. Quaternization of Chitosan from Schiff Bases and Iodomethane/Iodoethane

**Scheme 6:**
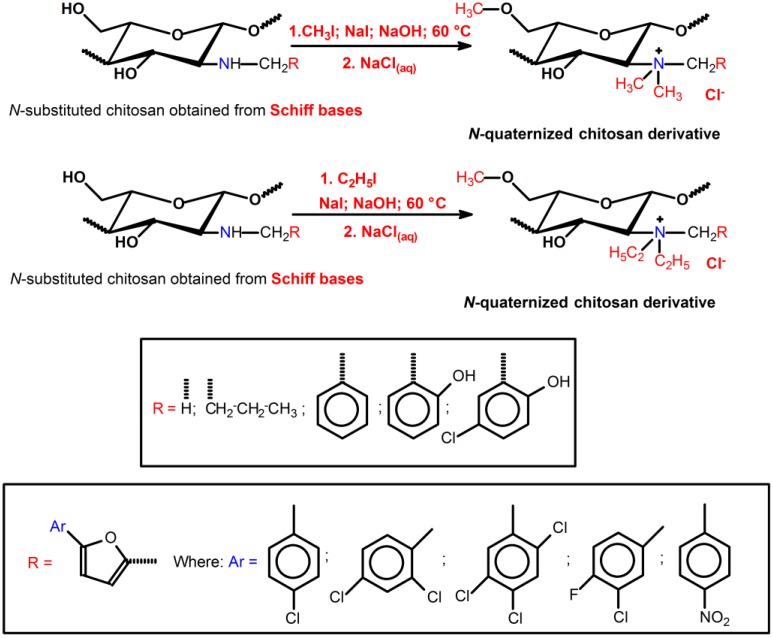
Route for synthesis of *N*-quaternized chitosan derivatives obtained from Schiff bases intermediates, followed by methylation with methyl iodide or ethyl iodide [65,78,79].

Other *N*-quaternized chitosan-derivatives can be synthesized from reductive alkylation using a series of different aldehydes via the formation of Schiff base intermediates, followed by methylation with methyl iodide or ethyl iodide (Scheme 6). Avadi *et* *al.* [79] and Sadeghi *et* *al.* [65] studied the antimicrobial activity of TMC and *N*,*N*-diethyl-*N*-methyl chitosan with high DQ. *N*,*N*-diethyl-*N*-methyl chitosan and TMC presented good bactericidal activity related to chitosan that was dependent on pH. Quaternary ammonium compounds have higher positive charge density than chitosan; their increased antibacterial effects can be attributed to the formation of polyelectrolyte complexes between the polymer and the negative peptidoglycans present on bacteria cell walls [65,79]. This interaction may in turn disrupt the cell wall and result in the inhibition of bacterial growth [65,79]. The bactericidal properties of TMC and *N*,*N*-diethyl-*N*-methyl chitosan at fixed DQ (50%) were compared. So, having the smaller alkyl groups, TMC showed a higher antibacterial effect against *S.* *aureus* than *N*,*N*-diethyl-*N*-methyl chitosan (DMCHT) [65,79]. According to Sadeghi *et* *al.* [65], the *N*-trimethyl group of TMC is smaller than the *N*-ethyl group, enabling easy reaction with the bacterial cell wall in comparison to the more voluminous *N*,*N*-diethyl-*N*-methyl groups of DMCHT derivative.

**Figure 3:**
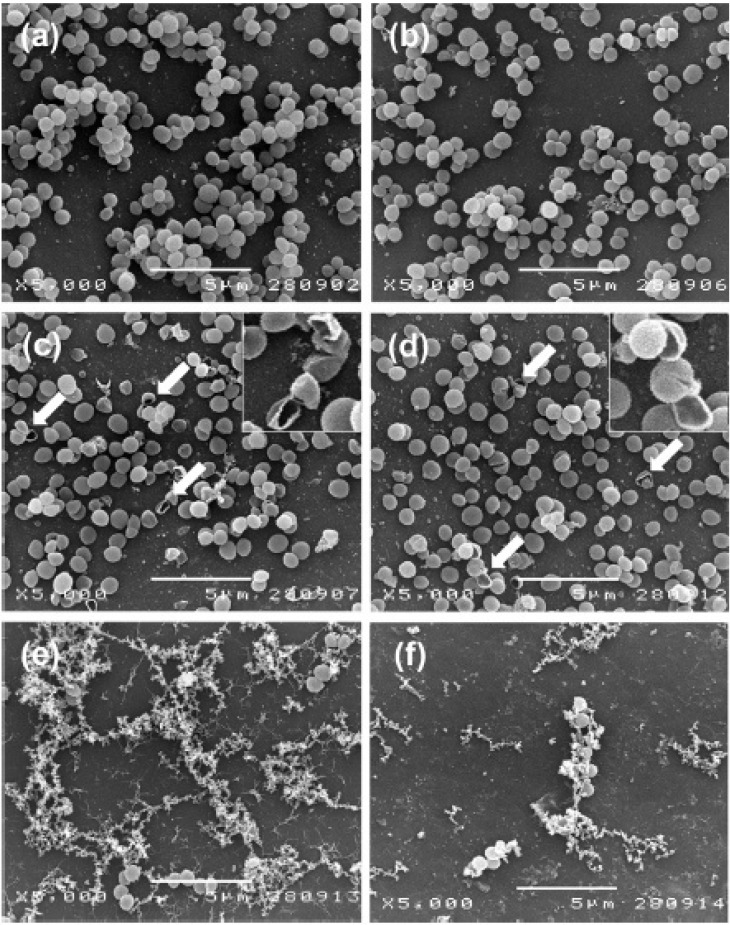
SEM micrographs of chitosan (**a**) and *x*BZDCHT films: 0.4BZDCHT (**b**); 0.8BZDCHT (**c**); 1.2BZDCHT (**d**); 1.6BZDCHT (**e**); and 2.0BZDCHT (**f**) after being incubated with the suspension of *S.* *aureus* for 24 h. The *x* term represents the amount of iodomethane utilized for chitosan-derivatives synthesis. Reprinted with permission from reference [78]. Copyright 2013 Elsevier.

The bactericidal activities of *N*-quaternized chitosan-derivatives based on DMCHT, *N*-benzyl-*N*,*N*-dimethyl chitosan (BZDCHT) and *N*-butyl-*N*,*N*-dimethyl chitosan (BDCHT) against *E.* *coli* and *S.* *aureus* were evaluated at pH 7.4 [78]. The antibacterial activities of these chitosan-derivatives were superior to those of chitosan. However, DMCHT and BDCHT derivatives exhibited greater antibacterial activities against both bacterial species than BZDCHT [78]. Amongst all the *N*-quaternized derivatives, it seems that greater hydrophobicity provided lower antibacterial activity, bearing in mind that all samples possess the same ζ potential [78]. In this case, the higher hydrophobic characteristic of *R* groups (*R* = hydrogen, phenyl, propyl among other as showed in the Scheme 6) seems to decrease the microbial activity of chitosan-derivatives [78]. The presence of hydrophobic bulky groups shields the interaction between *N*-quaternized sites and the microbial cell envelope, and reduces the bacteriostatic action. The influence of the chitosan-derivatives containing surface positive charge density on the antibacterial activity was examined against *S.* *aureus* on a series of BZDCHT films in which the charge magnitude was varied as a function of the iodomethane concentration utilized in the BZDCHT synthesis [78]. The increase of iodomethane concentration raised the DQ and consequently the BZDCHT films showed antimicrobial activity (Figure 3). The apparently damaged bacterial morphology (*S.* *aureus*) upon contact with the surface of the *N*-quaternized chitosan film (BZDCHT) was verified by SEM (Figure 3). The introduction of additional positive charges on the chitosan-derivative surface, via the versatile and simple process of heterogeneous quaternization (Scheme 6), significantly improves the antibacterial activity of the chitosan-derivative, especially in a neutral environment [78].

The antifungal activities of *N*-quaternized chitosan-derivatives such as BZDCHT, *N*-(2-hydroxyl-benzyl)-*N*,*N*-dimethyl chitosan (HBZDCHT) and *N*-(5-chloro-2-hydroxyl-benzyl)-*N*,*N*-dimethyl chitosan (CHBZDCHT*)* were evaluated against *Botrytis* *cinerea* Pers*.* and *Colletotrichum* *lagenarium* (Pass) Ell.et halst [50,51]. The results indicate that all *N*-quaternized chitosan-derivatives possess stronger antifungal activities than unmodified chitosan. Furthermore, *N*-quaternized derivatives with high molecular weight presented antifungal activities associated with the compounds with low molecular weight [50,51]. Chitosan containing substituted arylfurfural groups were obtained by heterocyclic modification through the formation of an intermediate Schiff base (Scheme 6) [28]. The results indicated that *N*-quaternized arylfuran chitosan-derivatives presented better antimicrobial activity related to unmodified chitosan [28]. According to Chetan *et* *al.* [28] another study confirmed that *N*-quaternized arylfuran chitosan-derivatives containing “Cl” and “NO_2_” in their backbone are effective for enhancing the antimicrobial activity of chitosan-derivatives (Scheme 6). The bactericidal activity of the *N*-quaternized arylfuran chitosan-derivatives (QACHT) containing heterocyclic aromatic substituents at 1000 ppm follows the order dichloride-QACHT > chloride-QACHT > trichloride-QACHT > nitre-QACHT > chloride-fluoride-QACHT (Scheme 6). The negative charge on gram-negative bacteria cell surfaces, higher than on gram-positive bacteria, leads to higher adsorption of *N*-quaternized arylfuran chitosan-derivatives and higher inhibitory effects against gram-negative bacteria [28]. The antifungal activity and antibacterial action of *N*-quaternized arylfuran chitosan-derivatives are similar. The antifungal mechanism also occurs due to the interaction between the cationic chains and the fungal cell surface containing negatively charged residues of macromolecules, leading to a leakage of intracellular electrolytes [28].

The antifungal activity of chitosan-derivatives can be improved by increasing the amount of quaternary ammonium moieties [52,53]. By increasing the number of ^+^NT sites, an increase in both solubility and interaction with the cell envelope will occur, increasing the antimicrobial activity as compared to chitosan. According to Chethan *et* *al.* [28] and Tan *et* *al.* [5] the antifungal activity tended to intensify with the increase in molecular weight, DQ and hydrophobic moiety containing substituted aromatic groups. On the other hand, the antifungal activity also depends on the fungus and bacteria types as well as on quaternization degree and chemical structure of the *N*-quaternized chitosan-derivatives [5].

**Scheme 7:**
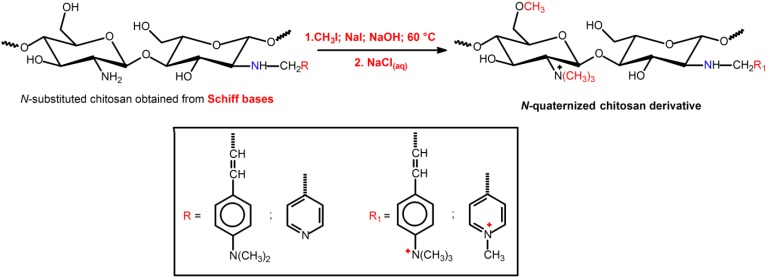
Route for synthesis of *N*-(4-*N*,*N*,*N*-trimethylcinnamyl) chitosan (TMCMCHT) and *N*-(4-pyridylmethyl) chitosan (PyMCHT) derivatives, containing *N*,*N*,*N*-trimethyl ammonium moieties in its structure [46,52,61,63].

Sajomsang *et* *al.* [46,52,61,63] studied the antimicrobial activity of TMC, *N*-(4-*N*,*N*,*N*-trimethylcinnamyl) chitosan (TMCMCHT) and *N*-(4-pyridylmethyl) chitosan (PyMCHT) derivatives containing *N*,*N*,*N*-trimethyl ammonium moieties in their structure (Scheme 7). These derivatives presented high positive charge density and strong bactericidal action at pH 7.2. It was found that TMCMCHT showed higher antibacterial activity than TMC, while PyMCHT exhibited reduced antibacterial activity against *E.* *coli* (ATCC 25922) and *S.* *aureus* (ATCC 6538) at the same DQ level [46,52,63]. The result showed the *N*,*N*,*N*-trimethyl ammonium group presented higher bactericidal activity than the *N*-methylpyridinium group at similar DQ and molecular weight. The resonance effect of the positive charge in the pyridine ring reduces the antibacterial activity of the *N*-methylpyridinium group [46,52]. So, the addition of the quaternary ammonium moiety on the amino groups of the chitosan-derivative was not necessarily enough to obtain antimicrobial action [52,61]. The key issue was the optimal positioning of the positive charges related to the polymer backbone [52,61]. In comparison to each of the chemical structures, it was found the antibacterial activity was not only dependent on the DQ, but also on the localization of positive charges and the molecular weight of chitosan-derivatives [61,63].

### 3.3. Quaternization of Chitosan Using Glycidyl Trimethylammonium Chloride

**Scheme 8:**
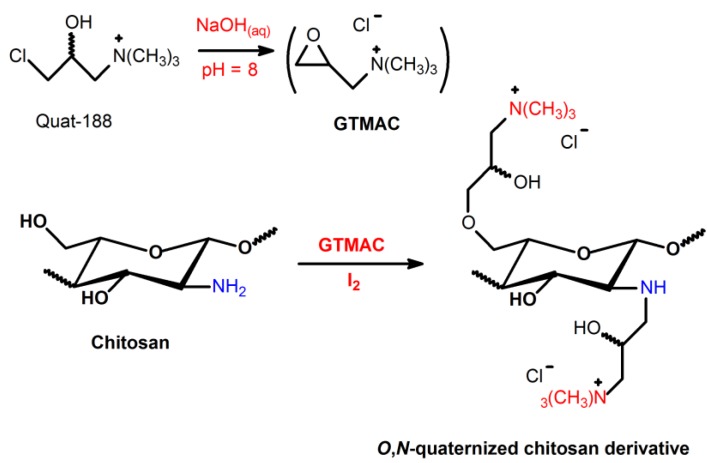
Route for synthesis of *O*,*N*-(2-hydroxy)propyl-3-trimethylammonium chitosan (*O*,*N*-quaternized chitosan derivative) [46,62].

Glycidyl trimethylammonium chloride (GTMAC) can be used as quarternizing agent [5,80,81,82,83]. When a primary amino group of chitosan reacts with GTMAC, the chains of the chitosan-derivative obtained are longer when associated to respective TMC [83]. In this case, the complete *N*-monoalkylation can be performed in water at 60 °C during 15 h [46]. The whole DQ was obtained from a molar ratio of 6:1 of GTMAC:GlcN of chitosan [46]. Under basic conditions, Daly and Manuszak-Guerrini synthesized the compound *N*-(2-hydroxy)propyl-3-trimethylammonium chitosan chloride from 3-chloro-2-hydroxypropyl trimethylammonium chloride (Quat-188 salt) (Scheme 8) [46]. Under basic conditions, Quat-188 readily generated the corresponding epoxide and quaternary moieties are introduced into chitosan chains by the reaction with the primary amine and hydroxyl groups via nucleophilic substitution [46,83]. The pH of the reaction condition was controlled (pH = 8) at room temperature for 48 h and iodine was used as a catalyst [46,62]. The full *N*-quaternization can be obtained at 50 °C for 24 h. The *O*-quaternization process also should occur at room temperature when the pH is adjusted to 8 (Scheme 8) [46,56,61,62]. The water-soluble *N*-(2-hydroxy)propyl-3-trimethylammonium chitosan chloride can be prepared by the reaction of chitosan and GTMAC. Bu *et* *al.* [84] used this potential side reaction and the as-obtained chitosan-derivative was used to modify cotton fabrics for improving aqueous pigment-based inkjet printing and antibacterial properties.

The hydroxyl groups inserted into *O*,*N*-(2-hydroxy)propyl-3-trimethylammonium chains give greater polarity to the derivative in relation to TMC. This effect may increase the antimicrobial activity, especially, if a gram-negative class of bacterium is taken into consideration. Some research groups synthesized *N*-(2-hydroxy)propyl-3-trimethylammonium and *O*,*N*-(2-hydroxy)propyl-3 trimethylammonium chitosan chloride and studied the antimicrobial activity of these compounds [52,62,85]. The mechanism of action is the same as that reported for chitosan, however the *O*,*N*-quaternized compounds obtained from Quat-188 (Scheme 8) presented higher positive charge density and solubility at any pH range. These features increased the applications spectrum of these derivatives, especially when it requires materials with good antimicrobial activity at pH close to the physiological condition [5,62,85].

### 3.4. Quaternization of Chitosan from Schiff Bases and Glycidyl Trimethylammonium Chloride

**Scheme 9:**
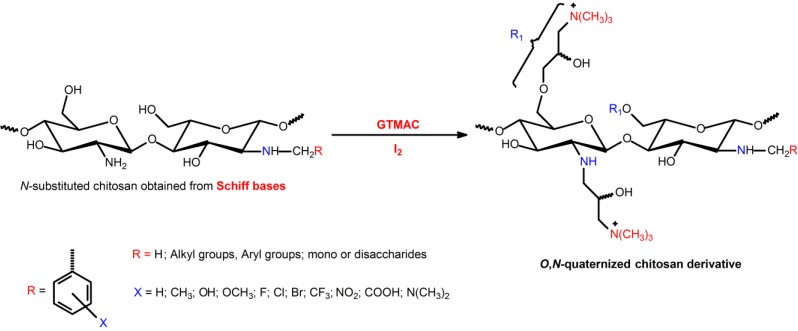
Route for synthesis of *O*,*N*-quaternized chitosan-derivatives containing different *N*-aryl substituents in their structures [86].

Sajomsang *et* *al.* [86] synthesized several chitosan-derivatives consisting of a variety of *N*-aryl substituents bearing both electron-donating and electron-withdrawing groups and chitosan-derivatives containing monosaccharide and disaccharide moieties. The synthesis was successfully performed by reductive *N*-alkylation from Schiff bases, and then the *O*,*N*-quaternization was obtained using Quat-188 (Scheme 9). The resulting quaternized compounds were water soluble at neutral condition (pH ≈ 7) [86].

Antimicrobial studies and the determination of minimum inhibitory concentration (MIC) of these materials were evaluated against *E.* *coli* (ATCC 25911) and *S.* *aureus* (ATCC 29113) bacteria (at pH 7.0) in order to investigate the biological activities of the chitosan-derivatives, which are dependent mainly on the extent of *N*-substitution. It was shown that the *N*-substitution obtained from Schiff bases was less than 10%, the chitosan-derivatives containing hydrophobic substituents (such as benzyl) presented low MIC values, whereas that the quaternized chitosan-derivatives obtained only from Quat-188 showed higher MIC values [86]. In this case, it was noted that neither electron-donating nor electron-withdrawing groups on the benzyl substituent affected the antibacterial activity against both type of bacteria. According to the authors, the presence of a hydrophilic moiety such as mono or disaccharides decreased the antimicrobial activity compared to the hydrophobic moiety as, for example, the *N*-benzyl group [86]. However, their antibacterial activities decreased with increasing *N*-substitution (>10%) due to the low quaternary ammonium moiety content. So, in order for the chitosan-derivatives (Scheme 9) showing significant bactericidal activity, these should hold up to 90% of saccharide residues containing *O*,*N*-quaternized moiety and still 10% of *N*-substituted groups with hydrophobic moiety (such as benzyl) in the backbone. All quaternized derivatives (Scheme 9) showed very low MIC values against both types of bacteria, which were in the range of 8–256 µg mL^−1^ [86]. The results clearly demonstrate that the hydrophobicity of *N*-substituted groups, obtained from Schiff bases, and the cationic charge density, introduced from Quat-188, play an important role in determining the antibacterial activity of quaternized chitosan-derivatives.

Mohamed *et* *al.* [62] introduced quaternary ammonium moieties into carboxymethyl chitosan containing electron-donating and electron-withdrawing groups on the benzyl substituent introduced on carboxymethyl chitosan chains from Schiff base reduction (Scheme 9). Keeping the DQ on the carboxymethyl chitosan backbone constant, the antimicrobial activity of *N*-quaternized carboxymethyl chitosan derivatives was affected not only by the nature of the microorganisms but also by the nature, position and number of the substituent groups on the phenyl ring [62]. Thus, the derivatives possessing groups of an electron-withdrawing nature showed higher bactericidal inhibition (with lower MIC values) than those having electron-donating groups on the *N*-quaternized carboxymethyl chitosan (CMCHT). According to Mohamed *et* *al.* [62] the antibacterial activities of these quaternized derivatives (4-nitro-CMCHT, 3-chloride-CMCHT and 3-bromine-CMCHT) against *E.* *coli* (RCMB 010052) were nearly equivalent to that of the standard drug Gentamycin. The greater antibacterial activity of the NO_2_-derivative related to the other compounds is due to the greater electron-withdrawing power of the nitro group. The electron-withdrawing groups such as NO_2_, CF_3_ Cl, Br and F make the nitrogen atom linked directly to the benzyl radical deficient in electrons, enhancing the antimicrobial activity of the derivatives [62].

**Scheme 10:**
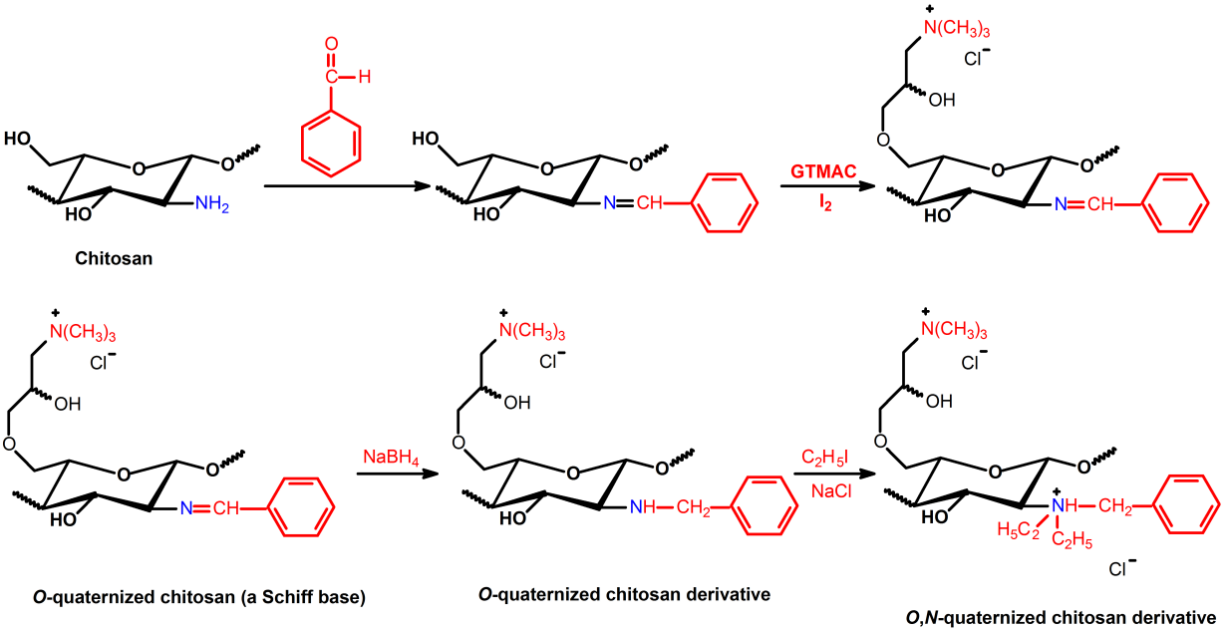
Route for synthesis of *O*,*N*-quaternized chitosan-derivatives performed by reductive *N*-alkylation from Schiff bases, then of *O*-quaternization from GTMAC and *N*-quaternization from ethyl iodide reducing agent [85].

Fu *et* *al.* [85] promoted the *N*-quaternization of the nitrogen atom attached to the benzyl radical by reaction with ethyl iodide in the presence of base and sodium iodide at 36 °C (Scheme 10). In this case, the *O*,*N*-quaternized derivative presented large amounts of quaternary ammonium chloride moieties, a fact that favored the interaction with negative residues at the bacterial cell surface, increasing the bactericidal effects mainly on gram-positive bacteria. As typical gram-positive bacteria, *S.* *aureus* cell walls contain negative substances, such as proteins, teichoic acid and lipopolysaccharides (LP) [85]. The antibacterial activity is achieved by the interaction among cationic ^+^N(CH_3_)_3_ groups (or ^+^NT sites) with the bacteria cells. This fact, affects the cell integrity by undermining the role of cell plasma membrane permeability of barriers, leading to loss of cell nutrients [85]. For the gram-negative bacteria *E.* *coli*, a thick layer of extracellular-type LP can prevent the entry of foreign macromolecules. The antibacterial effect on *E.* *coli* may be attributed to the synergistic actions of the quaternary ammonium group. The fabric treated with only *O*-quaternized chitosan derivative groups showed a slight decrease in antimicrobial activity on *E.* *coli*, compared to the *O*,*N*-quaternized chitosan derivatives because of the electron-donating effect of *N*-benzyl being reduced [85] due to the *N*-quaternization process (Scheme 10).

### 3.5. Quaternization of Chitosan from Glycidyl Trimethylammonium Chloride and Iodomethane

**Scheme 11:**
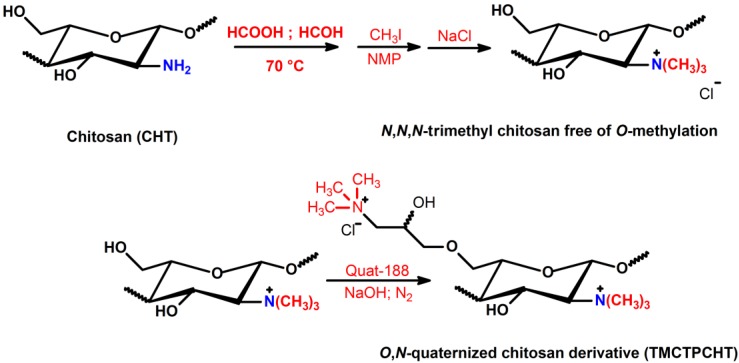
Route for synthesis of *O*,*N*-quaternized chitosan-derivatives performed by reductive *N*-alkylation of TMC free of *O*-methylation, then of *O*-quaternization from GTMAC reducing agent [87].

*N*,*N*,*N*-trimethyl *O*-(2-hydroxy-3-trimethylammonium propyl) chitosan derivative (TMCTPCHT) with different *O*-substitution degrees (DS) was synthesized by reacting TMC free of OM sites with Quat-188 [87] (Scheme 11).

The antibacterial activities of the quaternary ammonium chitosan-derivatives were compared to that of unmodified chitosan. Antibacterial activities against *S.* *aureus* (ATCC 6538) and *E.* *coli* (ATCC DH5α) under weakly basic (pH 7.2) and weakly acidic (pH 5.5) conditions were evaluated. In this case, at pH 5.5 the quaternary ammonium derivatives exhibit antibacterial behavior and all the chitosan-derivatives have stronger antibacterial activity compared to chitosan [87]. After further *O*-quaternization, TMCTPCHT exhibited stronger antibacterial activity than TMC and the activity of TMCTPCHT became higher, at both pH 5.5 and 7.2, with the increase of DS. To carry out the quaternization process, modifications with flexible chitosan backbone side chains are more suitable for enhancing the interaction between chitosan-derivatives and the cell envelope [87]. The antibacterial activity of chitosan and respective quaternary ammonium derivatives are stronger under weakly acidic conditions than under weakly basic conditions. However, this fact depends on the DQ and DS at the C6 position (Scheme 11). According to Xu *et* *al.* [87], chitosan and its quaternary ammonium derivatives exhibit stronger antibacterial activity against *S.* *aureus* (ATCC 6538) than against *E.* *coli* (ATCCDH5α). This can be attributed to the different cell envelopes of *S.* *aureus* and *E.* *coli*. Chitosan and its quaternary ammonium derivatives have greater difficulty penetrating the outer membrane of *E.* *coli*.

### 3.6. Quaternization of Chitosan through Other Methods

The low solubility of chitosan in organic solvents has proven to be one of the main challenges for the synthesis of new chitosan-derivatives. For example, the synthesis of *O*-methyl TMC is performed under heterogeneous conditions where the unmodified chitosan (starting material) is only partially solubilized in *N*-methyl-2-pyrrolidinone (NMP). These reactions often yield materials with large structural heterogeneity when compared to the starting material. To overcome this barrier, protection strategies have been introduced, allowing the occurrence of reactions that produce more uniform materials [76,88].

**Scheme 12:**
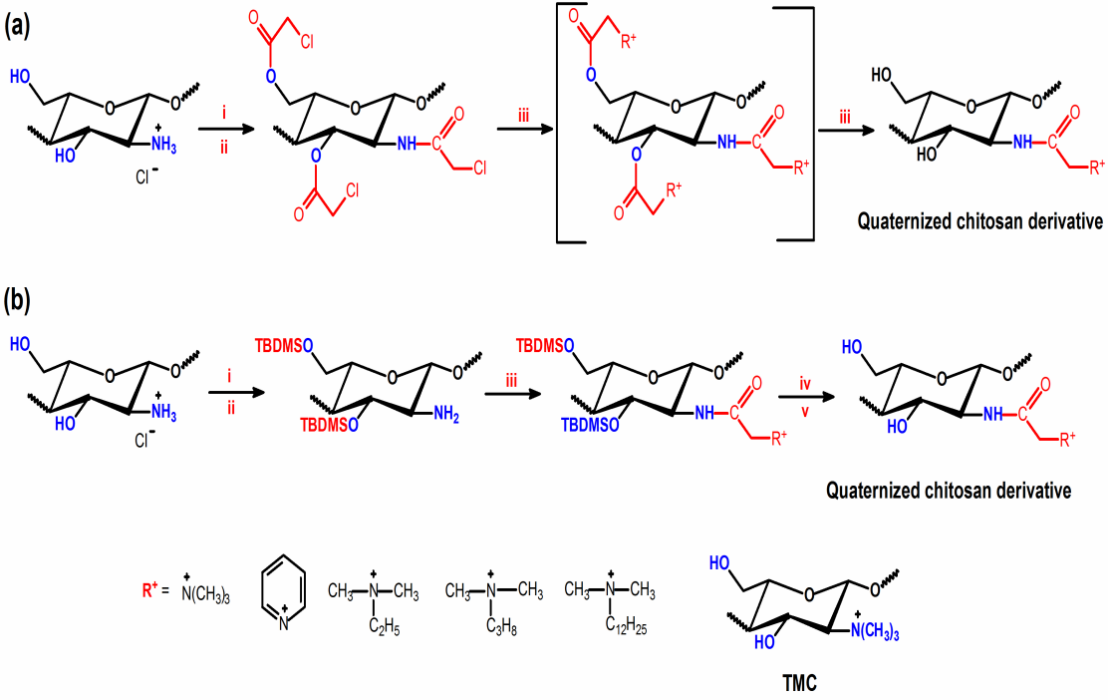
Route for synthesis of the *N*-quaternized chitosan derivatives. Reagents and conditions: (**a**) (**i**) TEA, pyridine, H_2_O; (**ii**) chloracetyl chloride, TEA, DMF, N_2_ atmosphere, 72 h, 22 °C; (**iii**) tertiary amine, DMF or NMP or pyridine, N_2_ atmosphere, NaI as catalyst when TEA and tripropylamine as reagents, 72 h, 60 °C, ion exchanged, dialysis; and (**b**) (**i**) methansulfonic acid, H_2_O; (**ii**) TBDMSCl, imidazole, DMSO, N_2_ atmosphere, 0 °C, 20 min, 2 × 24 h, 22 °C; (**iii**) chloracetyl chloride, TEA, pyridine dichloromethane, 1 h, 0 °C; (**iv**) dimethyldodecylamine or dimethylbutylamine, chloroform, 40 h, 22 °C; and (**v**) concentration of HCl, ethanol, 6 h, 22 °C [76,88].

Quaternary chitosan-derivatives were synthesized by Benediktsdottir *et* *al.* [76] and by Runarsson *et* *al.* [88] for the purpose of investigating the structure activity relationship for the antibacterial effect. Novel methods using protection strategies were used in such synthesis (Scheme 12). The chitosan-derivatives can be synthesized by two steps from protection of the hydroxyl groups. However, three reaction steps were performed, starting from 3,6-*O*-di-*tert*-butyldimethylsilyl chitosan (3,6-*O*-di-TBDMS chitosan) as intermediate compound to obtain chitosan-derivatives with the bulky *N*,*N*-dimethyl-*N*-dodecyl and *N*,*N*-dimethyl-*N*-butyl side chains [88] (Scheme 12).

The MIC values for the all derivatives exposed in Scheme 12 ranged from 8.0 × 10^−3^ to 8.2 g L^−1^. In this case, the antimicrobial action was evaluated using the TMC as reference [88]. The *N*-(2-(*N*,*N*-dimethyl-*N*-dodecyl ammonium) derivatives were less active when compared to the compounds containing *N*-(2-*N*,*N*,*N*-trimetylammonium) or *N*-(2-(*N*-pyridiniumyl)) quaternary moiety in their structures (Scheme 12). The studies indicated that TMC was the most active compound [88]. According to Runarsson *et* *al.* [88] the position of the quaternary group is important for the antibacterial action of chitosan-derivatives. Furthermore, the presence of bulky groups with a hydrophobic character linked to the quaternary nitrogen atom considerably reduces microbial activity [88].

## 4. Antimicrobial Activity of *N*-Quaternized Chitosan Derivative-Based Materials

The literature reports that water-soluble chitosan-derivatives exhibit appreciable antimicrobial activity in aqueous solution. Previously, the excellent antimicrobial properties of such derivatives were presented, highlighting those chitosan-derivatives containing *N*-quaternized groups in their structures. The higher DQ of chitosan-derivative increases its solubility. On the other hand, chitosan based-materials have been restricted to the application of antimicrobial properties such as beads, films, fibers and particle-based chitosan or chitosan-derivative materials aimed at biomedical applications [2,35,65,89,90]. The shift of physical state could bring about significant changes in the biocide activity of the materials, whereas chitosan and their derivatives presented good antimicrobial activity, related to the compounds in the solid state, such as films, particles and fibers [2]. When the pH of the environment is higher than that of the p*K*_a_ value of chitosan, chelating effects and hydrophobic characteristics of chitosan are the factors responsible for the antimicrobial activity instead of electrostatic forces [2]. So, chitosan can present inhibitory activity by chelation of the metallic cations present in cell walls. However, chitosan-derivatives containing lipophilic groups display antibacterial activities through hydrophobic and chelation effects. These two effects explain why the chitosan-derivatives free of *N*-quaternized sites in their backbone showed higher antimicrobial activity, compared to unmodified chitosan under neutral or higher pH conditions [2].

On the other hand, the high zeta potential and good contact surface of *N*-quaternized chitosan derivative-based materials are factors that significantly increase the bactericidal activity [2]. The positive ζ potential values of chitosan-derivatives are due to the portion of the chitosan-derivatives that are protonated and disassociated in solution [2,65]. For example, TMC nanoparticles crosslinked with tripolyphosphate anion with positive ζ potential showed good antibacterial inhibition against *S.* *aureus* and *S.* *epidermidis* [65]. In this case, TMC nanoparticles (TMC-NP) exhibit better antibacterial activity when compared to chitosan nanoparticles (chitosan-NP). The average ζ potential in water of TMC-NP (suspension of concentration 1.0 mg L^−1^) was 22 mV, while for chitosan-NP (suspension of concentration 1.0 mg L^−1^) was 15.9 mV. In this case, TMC-NP presented better antimicrobial activity related to chitosan-NP, due to the presence of the quaternary ammonium groups (DQ = 50 ± 5%) [65]. However, the TMC presented high average ζ potential (43.2 mV) and antimicrobial assays suggested that the polymers in free form (1.0 mg L^−1^), *i.e.*, in aqueous solution, showed higher antibacterial activity against gram-positive bacteria than TMC-NP [65].

*N*-quaternized chitosan-based films have been developed and the process aims at new biomaterials with antimicrobial, anti-adhesive and mechanical properties [2,91]. Follmann *et* *al.* [19] developed multilayer thin films based on TMC/heparin (TMC/HP). TMC with different DQs (20% and 80%) self-assembled with heparin were prepared at pH 3.0 and 7.4. The initial adhesion test of *E.* *coli* (ATCC 26922) on TMC/HP surfaces showed effective anti-adhesive properties. On the other hand, the *in* *vitro* antimicrobial test showed that the TMC/HP multilayer films based on TMC80 (multilayer film obtained from *O*-methyl TMC with DQ = 80%) can kill the *E.* *coli* bacteria at pH 7.4 [19]. Therefore, anti-adhesive and antibacterial self-assembled films may have good potential for coatings and surface modification of biomedical applications [19]. *N*-[(2-hydroxyl-3-trimethylammonium)propyl] chitosan chloride (HTACC) exhibits antibacterial activity, as soluble in liquid (solution) and in solid, and as coated onto others materials [2,91]. Graisuwan *et* *al.* [91] obtained thin films based on HTACC assembled with poly (acrylic acid). In this case, the multilayer film containing HTACC moieties exhibited moderate antibacterial activity against *E.* *coli* and *S.* *aureus* [91].

Electrospinning is an ideal technique for producing polymer fibers with diameters down to nanoscale range and micro- and nanofibrous materials are appropriate for obtaining wound dressings [2,92,93]. Because of their excellent properties (good porosity, good surface area and diameters in nanoscale), electrospun fibers formed from ultrafine polymeric fibers have attracted great attention [2]. Nanofibres of poly (vinyl alcohol) [92] and poly (vinyl pyrrolidone) [93] coated with TMC has been successfully prepared using the electrospinning technique. For example, TMC/poly (vinyl pyrrolidone) nanofibres showed high antimicrobial activity against gram-negative bacteria (*E.* *coli*) and gram-positive bacteria (*S.* *aureus*) [93]. On the other hand, in a previous work, Ignatova *et* *al.* [92] showed that the antibacterial activity of TMC/poly vinyl alcohol fibres was bactericidal only against *S.* *aureus* (ATCC 749). Thus, the nanofibers coated with TMC (polycationic polymer) mats are promising for wound-healing applications [93].

## 5. Future Trends for Antimicrobial Applications of Chitosan-Based Materials

Chitosan is: (i) Obtained through deacetylation from chitin, the most abundant polysaccharide in the world after cellulose and thus obtainable from renewable sources; (ii) Capable of being modified using multiple alternatives for chitosan-chemical modifications that provide different desirable properties, such as good solubility at neutral and basic media; and (iii) Often, along with chitosan-derivatives, capable of significant antimicrobial activity, most of them often biocompatible; this makes chitosan, chitosan-derivatives and materials based on chitosan or chitosan derivatives excellent material alternatives for application as antimicrobial agents in different developed fields such as the food [94], medical [95], textile [96] and paper-making [97] industries, among others. For instance, the film-forming property of chitosan and chitosan-derivatives combined with the antimicrobial activity is a very important issue in producing edible coatings for preserving fruits, and natural or industrialized foods. Campos *et* *al.* [94] published a review in which the main hydrocolloids and antimicrobials used for building up edible films and coatings, the methods used to estimate the antimicrobial activity, the applications and the legislation concerning edible films and coatings are deeply described. Chitosan and chitosan-derivatives are clearly pointed out by these authors as important antimicrobial agents and they anticipate that the demand for them will certainly increase in the coming decades in response to consumer demand for greener additives [98]. Consumer demands for more natural foods, and also for environmental protection, induce the development of new packaging materials possessing antimicrobial agents. Engler *et* *al.* [95] provided a review in which synthetic macromolecular antimicrobials are considered to be a highly promising class of therapeutics with great potential for combating multidrug-resistant microbes. Quaternized chitosans, including TMC, were highlighted by these authors as very important chitosan derivatives with immense potential for the future. Polysaccharide-based biomaterials with antimicrobial and antioxidant properties were reviewed by Coma [99]. Modification of chitosan leading to different chitosan-derivatives, changing the morphology from flat films to fibrous surfaces, and changing porosity, wettability and other characteristics of chitosan and chitosan-derivatives, allowing the production of implants and biomedical devices with higher resistance to microbial adhesion and good capability for biofilm formation [100], support a good scenario for the future.

Given its doubtless potential in the field, the following can be stated: systems based on “release on command”, in which an antimicrobial agent is released at the same time as the microbial growth occurs, will be disseminated in the future on antimicrobial materials associated to high technology. The concept of this technology is that when any change in the medium such as pH, temperature or UV light occurs (due to initial microbial action), the antimicrobial system responds promptly and accordingly. Therefore, such a system turns active only in response to very specific conditions. This type of system is based on bioactive compounds, where release is triggered at the time and place where needed. In the case of antimicrobial agents, the consensus is that they will be released if one or more type of bacterial growth occurs, thus inhibiting growth of the emerging bacteria. This would, of course, enable a decrease in the active agent concentrations needed for effective effects.

## 6. Concluding Remarks

*N*-quaternization and/or *O*,*N*-quaternization are the most used ways to modify chitosan resulting in a water-soluble chitosan-derivative in a wide pH range including neutral and basic conditions. Such chitosan-derivatives present excellent antimicrobial activity due to permanent positive charges on the polymer backbone. The antimicrobial activity provided by chitosan, chitosan derivatives and chitosan-based materials is especially relevant. Various wide-ranging antimicrobial chitosan, chitosan-derivatives and chitosan-based materials exhibiting potent antimicrobial activity through membrane-lysis mechanisms have been described in this review. The uses of antimicrobial agents such as chitosan and chitosan-derivatives are likely to grow steadily in the future because of greater consumer demands for materials (foods, paper, textiles, biomaterials, *etc.*) with minimal possibility of microbial infections. New studies to enlarge the number of chitosan-derivatives with still higher antimicrobial effects are needed. In summary, this review is an attempt to show that chitosan, chitosan-derivatives and chitosan-based materials are value-added if the target is the developing of antimicrobial agents for use in different technological fields, providing modern society with new prospects for healthy aging and a higher quality of life.
